# Sea level anomalies affect the ocean circulation at abyssal depths

**DOI:** 10.1038/s41598-023-48074-9

**Published:** 2023-11-27

**Authors:** D. I. Frey, E. G. Morozov, D. A. Smirnova

**Affiliations:** 1 Shirshov Institute of Oceanology, Russian Academy of Sciences, Moscow, Russia; 2 Marine Hydrophysical Institute, Russian Academy of Sciences, Sevastopol, Russia; 3 Moscow Institute of Physics and Technology, Dolgoprudny, Russia

**Keywords:** Physical oceanography, Physical oceanography, Fluid dynamics

## Abstract

Abyssal channels are the key points controlling bottom circulation of the World Ocean. They provide meridional transport of the coldest Antarctic Bottom Water between deep-water basins influencing the meridional overturning circulation and the climate on a global scale. Here we show that the synoptic variability of deep-water flows including blocking abyssal currents between deep ocean basins is related to sea level anomalies observed over the channels. Our results demonstrate that processes at the ocean surface have a more significant connection with the bottom circulation than it was considered earlier. This study opens a discussion of the importance of mesoscale eddies and air-sea interactions on water exchange between abyssal basins, meridional heat transport in the ocean, and possible responses of the ocean to the observed sea level rise in a changing climate.

## Introduction

Abyssal currents as well as their temporal variability remain the less studied part of the World Ocean circulation: they are invisible from satellites, not covered by most autonomous ocean platforms, and rarely simulated by numerical models. Thus, most studies of deep-water circulation rely on very expensive and time-consuming observations from research vessels. Unlike surface currents, the abyssal circulation is significantly influenced by bottom topography: deep-water passages, channels, troughs, and fracture zones are the key points providing the transport of Antarctic Bottom Water (AABW) between ocean basins. Monitoring of bottom currents at these key points provides data on abyssal water exchange between deep ocean basins^1–6^. Significant variability of the bottom currents at the synoptic time scale was documented almost in all studied abyssal channels^7–12^.

**Figure 1:**
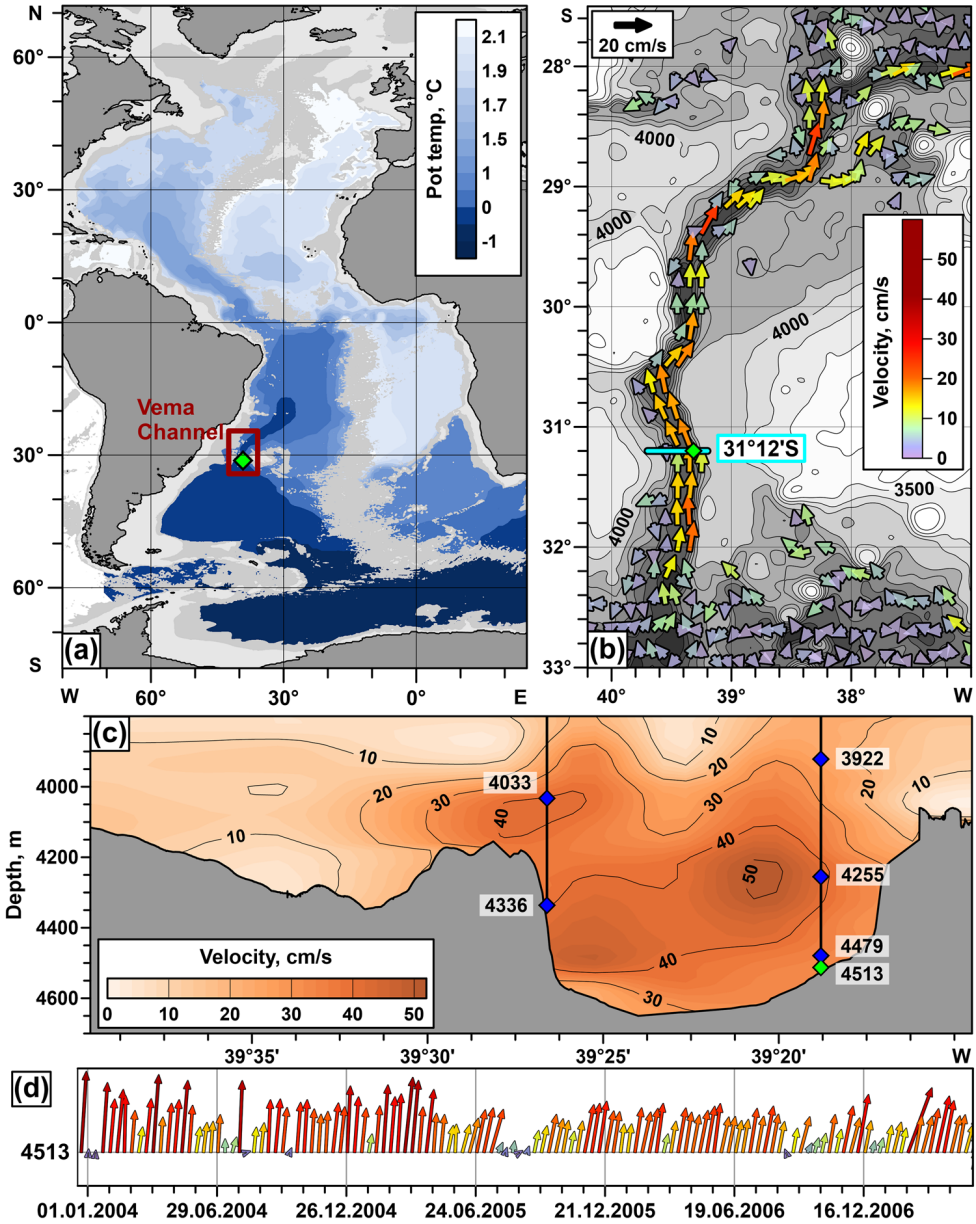
The Vema Channel as the key point controlling bottom water temperature in the Central and North Atlantic. (**a**) Distribution of bottom potential temperature in the Atlantic Ocean at depths greater than 3800 m according to WOA2018 data^21^. (**b**) Modeled mean bottom current velocities in the region of the Vema Channel connecting the Argentine and Brazil basins based on the data published in ref.^22,23^. (c) Along-channel velocity distribution at the section across the Vema Channel based on LADCP data. Locations of two moorings are shown by black solid lines; the moored current meters are indicated by blue diamonds; the numbers indicate their depth in meters. (**d**) The bottom velocity time series measured in the channel from December 18, 2003 to May 18, 2007 from the instrument shown by green diamond in panel (**c**). The maps were generated using Golden Software Surfer version 18.1.186 (https://www.goldensoftware.com/products/surfer/).

In the Atlantic Ocean, the abyssal ocean layer is filled with cold and dense AABW defined as water with potential temperature below 2 °C^13,14^. This water mass occupies more than one-third of the entire volume of the World Ocean^15^; its northward propagation is a major component of the ocean thermohaline circulation; AABW affects the Earth climate on a global scale^14^. One of the most studied abyssal passages of the World Ocean is the Vema Channel connecting the Argentine and Brazil Basin in the Southwest Atlantic^16–18^ (Fig. 1). This channel was formed due to the long-term bottom erosion caused by the AABW flow^19^. It provides transport of the coldest AABW to the Central, Northwest, and Northeast Atlantic, thereby influencing the potential temperature of the major part of the Atlantic Ocean. Recent studies have documented an increase in AABW temperatures in the Vema Channel^20^; this signal is propagating from the Weddell Sea to the mid-latitudes of the Northern Hemisphere affecting bottom temperatures over the entire Atlantic Ocean^6,14^. Hence, we emphasize the importance of the Vema Channel on a global scale.

Our knowledge of the mean abyssal circulation is mainly based on measurements of thermohaline water properties rather than direct velocity observations. However, at narrow pathways like the Vema Channel, the kinematic structure of the bottom layer becomes more informative due to strong acceleration of the bottom flows. Properties of the bottom gravity current in the Vema Channel were repeatedly studied from the early 1980s^14^. The CTD measurements performed in the Vema Channel during the last 40 years indicated a linear warming trend of 0.0019 °C/year in the AABW layer. Almost the same warming trends are observed in the ocean abyss in different regions^24–26^. Lowered ADCP measurements showed near-bottom velocity up to 55 cm/s depending on the location^14^ (Fig. 1c). Numerical simulations confirm the presence of the high-velocity jet and provide information about its three-dimensional structure^22,27^ (Fig. 1b). Moored velocity observations carried out in 1979–1981, 1991–1992, 1998–2000, 2003–2007, and 2019–2020 show the existence of variability of bottom currents observed both in current velocities (Fig. 1d) and AABW potential temperatures^20,28,29^. However, the causes of this temporal variability remain poorly understood. In particular, the possible influence of mesoscale eddies on the dynamics of the gravity current in the Vema Channel has been never studied previously. The goal of this paper is to show that this variability is related to the sea level anomalies observed at the sea surface by satellite altimetry. This result complements works on abyssal circulation variability on different time scales from tidal and inertial motions^28^ to multidecadal time scale^20,30^.

## Results

In this study, we suggest a simple explanation of the bottom current variations in the Vema Channel including periodical blocking events, when the current almost completely stops. The main idea is that mesoscale eddies propagating over the Vema Channel change hydrostatic pressure over the entire water column. The hydrostatic pressure at the entrance to the channel is different from the pressure at the exit from the channel; this difference causes the abyssal pressure gradient that forces abyssal waters through the channel. It is natural to assume that variations in this pressure gradient lead to variations in the velocity of the abyssal current. Thus, changes in the abyssal pressure gradient, which can be related to cyclones and anticyclones at the sea surface, lead to acceleration or deceleration of the abyssal flows. It should be noted that the eddy kinetic energy is especially high in the Southwest Atlantic. These eddies are generated by the collision of the warm Brazil Current and cold Malvinas Current near the South American continental slope^31^. These eddies propagate over a wide area in the South Atlantic; some sea level anomalies reach the entrance to the Vema Channel at approximately 34°S. Here, we focused on the combined analysis of sea level anomalies and abyssal flows in this part of the Atlantic Ocean.

### Relation between sea level anomalies and bottom currents

**Figure 2:**
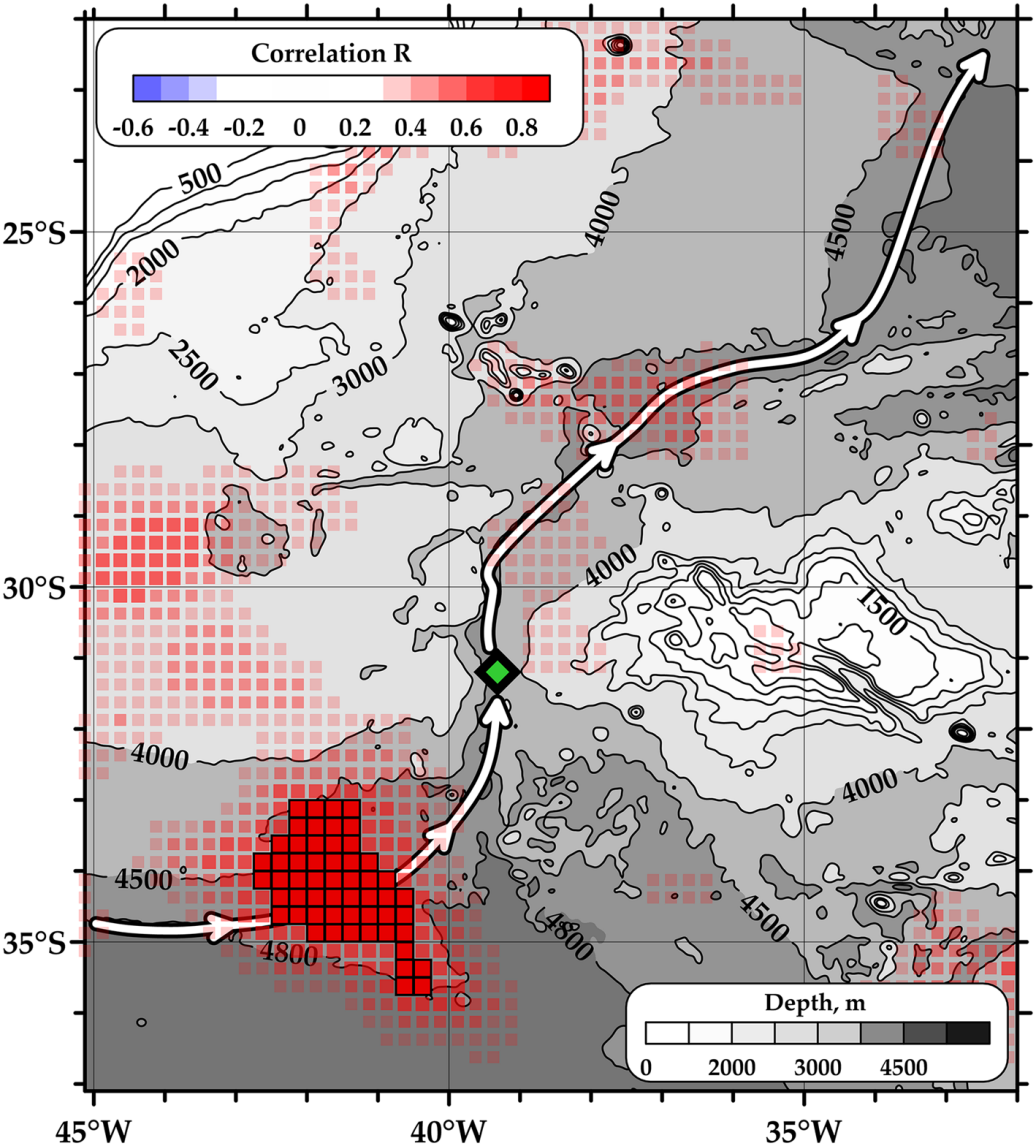
Spatial pattern of correlation between sea level anomalies and bottom velocities. The location of mooring is indicated by green diamond. The main pathway of the AABW propagation is shown by white arrows according to ref^14^. Only points with correlation exceeding 0.3 are shown; the points with correlation exceeding 0.6 are highlighted by red squares and thick black lines. The size of each grid point is 0.25° × 0.25° according to the gridded altimetry data resolution. The area of strong correlation coincides with the wide area between the 4500 m and 4800 m isobaths on the pathway of AABW spreading into the Vema Channel. The bottom topography is shown by shades of grey according to the GEBCO2022 database. The map was generated using Golden Software Surfer version 18.1.186 (https://www.goldensoftware.com/products/surfer/).

Despite the fact that the described idea of the relation between sea level anomalies and bottom flows is quite obvious, the spatial and temporal scales of this influence are not clear. That is why we combined daily satellite gridded altimetry maps^32^ over a wide area in the Southwest Atlantic and velocity measurements carried out at a mooring in the Vema Channel. The altimetry data have a spatial resolution of 0.25° and a daily temporal resolution; they contain information about sea level anomalies (SLA) relative to the mean sea level. The mooring was deployed at the core of the abyssal flow; the LADCP data shown in Fig. 1c illustrate how the current meters were located relative to the AABW jets. The data from the current meter closest to the bottom have been taken for the analysis; it was located at depths of approximately 4500 m. The recorders located at different depths show synchronous variations in the AABW flow velocity indicating that changes in the abyssal circulation affect the entire bottom layer. The velocities were measured with a temporal resolution of 2 h. The 1-day averaged velocity was taken for the analysis in order to equalize the temporal resolution of satellite altimetry and in situ velocity. Using these data, the correlation map between SLA and the AABW velocity was calculated (Fig. 2). At each point of the grid, the correlation was calculated between two time series: the SLA at this point and the AABW velocity measured at a mooring located in the Vema Channel (31.25° S, 39.32° W, green diamond in Fig. 2). The correlation coefficient is lower than 0.3 over the major part of the Vema Channel region. In particular, no relation has been observed between the AABW velocity and SLA variations directly above the mooring. Instead, the area of high correlation is located approximately 400 km south of the mooring at the entrance to the Vema Channel between 33° and 36° S and between 43° and 40° W (red squares in Fig. 2 show correlation exceeding 0.6 reaching a maximum of 0.69). This area follows the pathway of the AABW flow which is pressed to the deep slope of the Santa Catarina Plateau. The area coincides very well with the region between 4500 and 4800 m isobaths. The distance between these isobaths increases, the deep slope becomes flatter, the AABW flow probably slows down and the influence of sea level anomalies becomes more significant. The total area of high correlation between SLA and AABW velocities reaches 35,000 km^2^.

### Spatial structure of sea level anomalies

**Figure 3:**
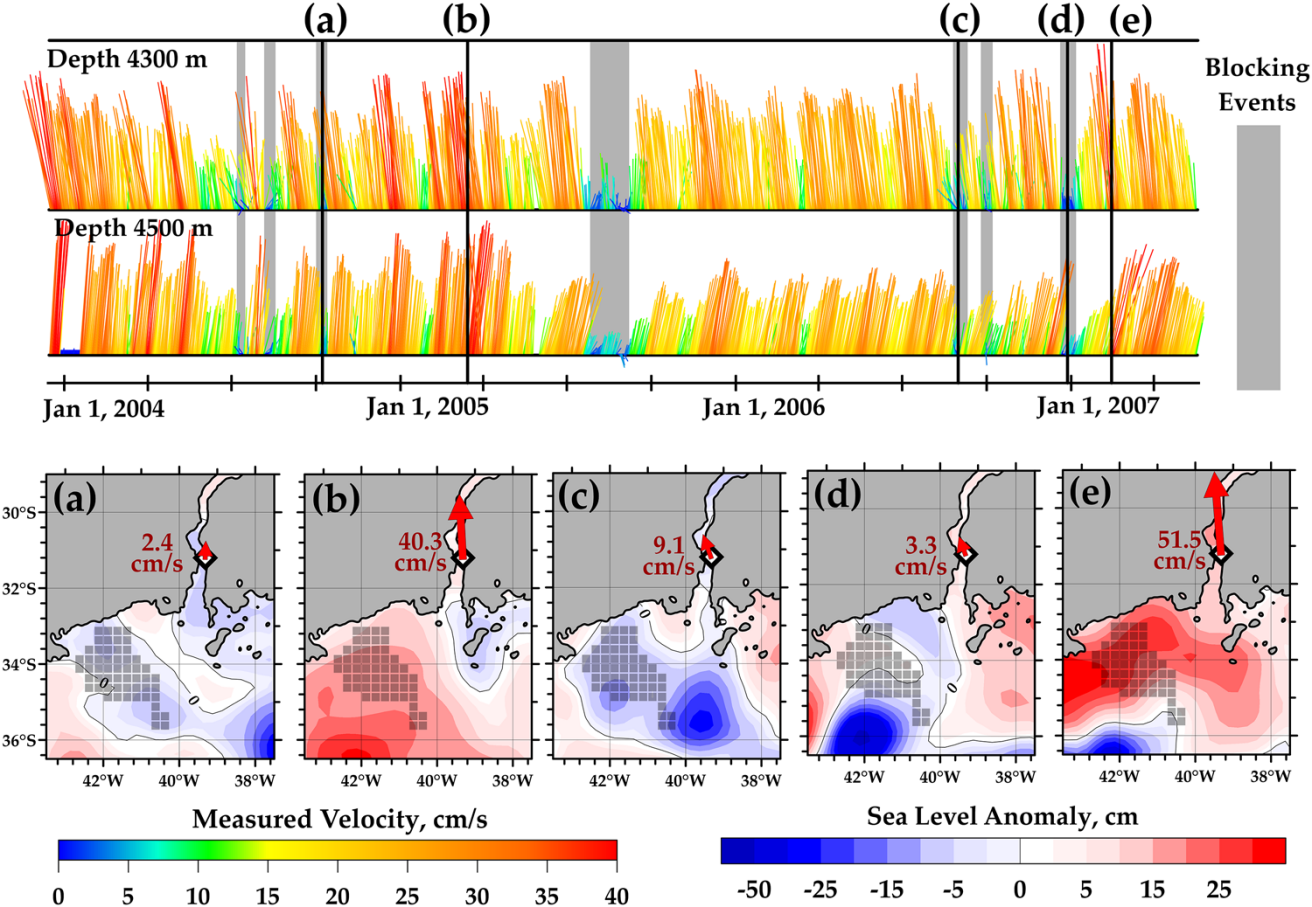
Blocking events in the Vema Channel. Velocity time series based on moored measurements at 4300 m and 4500 m depth are shown in the upper panel. Bottom panels show SLA distributions at several dates: October 8, 2004 (**a**), March 15, 2005 (**b**), August 31, 2006 (**c**), December 28, 2006 (**d**), and February 14, 2007 (**e**). These maps correspond to the short time periods when the abyssal currents are either slow (**a**, **c**, **d**) or fast (**b**, **e**). Gray squares indicate the area of high (more than 0.6) correlation between the abyssal velocity and SLA data. The white diamond in the bottom panels indicates the location of mooring. Ocean depths shallower than 4500 are shown by solid gray coloring. The maps were generated using Golden Software Surfer version 18.1.186 (https://www.goldensoftware.com/products/surfer/).

In order to evaluate spatial extents of studied sea level anomalies, the SLA maps are presented in several cases of extremely high and low velocities in the Vema Channel (Fig. 3). In these cases, maximum sea level anomalies vary between − 18 and 31 cm, the corresponding variations of the velocity at 4300 m layer range from 4 to 53 cm/s. The mean velocity at this level is 26.5 cm/s; 200 m deeper, the mean velocity is 20.4 cm/s. Velocities at these two levels vary synchronously indicating that sea surface anomalies affect the entire AABW layer. Thus, even a negative anomaly of 20 cm in the sea level at the entrance to the Vema Channel is related to blocking events that occasionally disconnect the Brazil Basin from the source of AABW. If we define the blocking events as the periods when the velocity does not exceed 10 cm/s, we get 8 events during 3.4 years of measurements in 2003–2007. These events last from 7 to 21 days. It should be noted that the entrance to the Vema Channel is a region of relatively low eddy kinetic energy compared to the most intense mesoscale activity regions of the Southwestern Atlantic^33^. The mesoscale eddies which cause the studied anomalies were quite weak. The extreme values of the sea level anomalies (− 18 and 31 cm) were low; for example, the cyclonic and anticyclonic eddies located at the Brazil-Malvinas Confluence zone at approximately 40° S, 55° W caused sea level anomalies up to 100 cm^33,34^. However, even mesoscale eddies of relatively low intensity at the entrance to the Vema Channel were related to the observed correlation.

### Spatial structure of the abyssal flow

**Figure 4:**
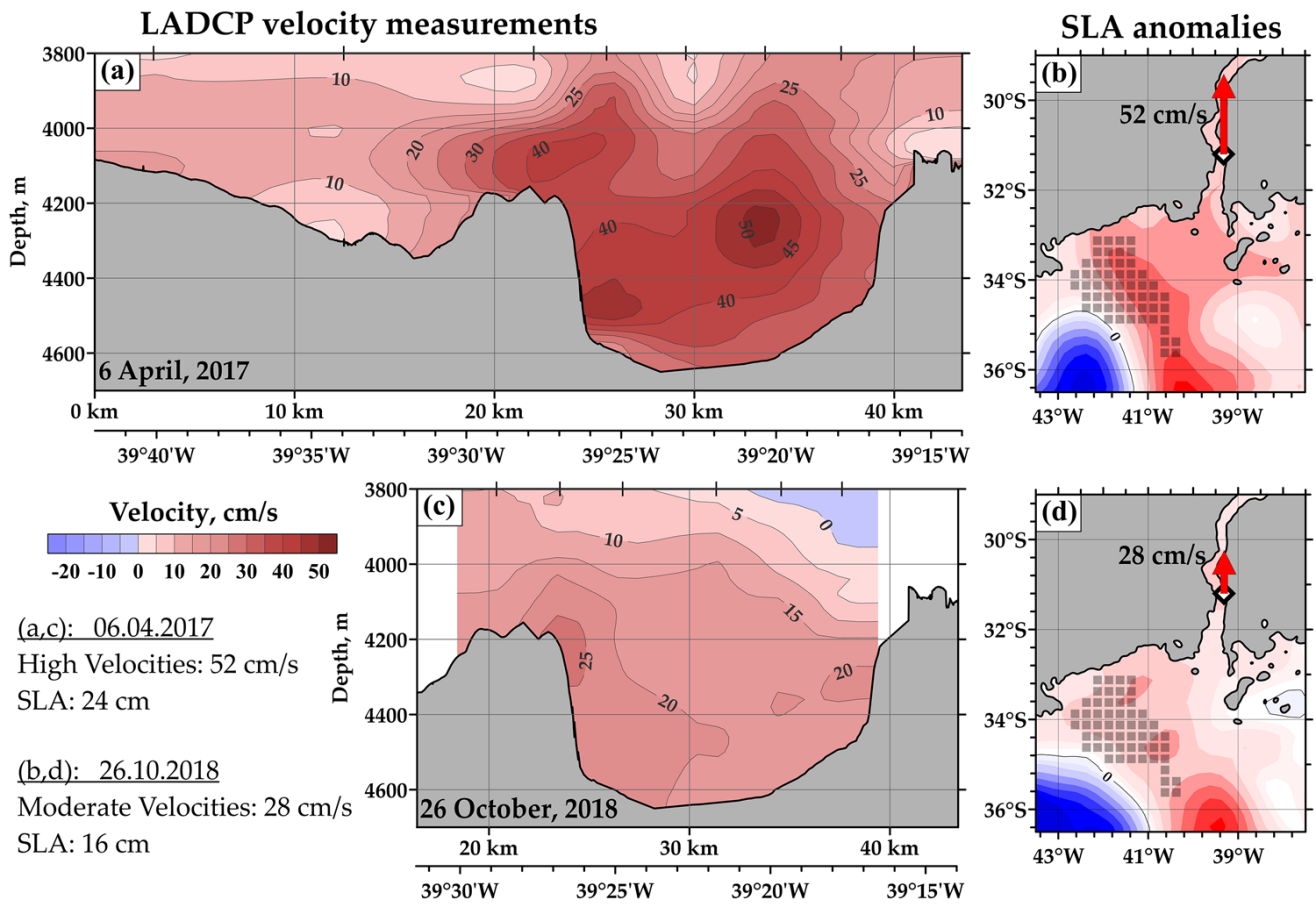
Spatial structure of the abyssal current in the Vema Channel. Distribution of along-channel measured LADCP velocity (**a**, **c**) and corresponding sea level anomalies (**b**, **d**) based on satellite altimetry. Two modes of the abyssal flow are presented: fast abyssal flow observed on April 4, 2017 (**a**, **b**), and moderate flow measured on October, 26 (**c**, **d**). The maximum measured velocities in these cases are 52 cm/s and 28 cm/s, respectively. Gray squares in panels (**b**, **d**) indicate the area of high (more than 0.6) correlation between the abyssal velocity and SLA data. The white diamond in the bottom panels indicates the location of mooring. Ocean depths shallower than 4500 m are shown by solid gray coloring in panels (**b**, **d**). In panels (**a**, **c**) bottom topography profile along the section is shown based on the Kongsberg EA600 echosounder survey. The maps were generated using Golden Software Surfer version 18.1.186 (https://www.goldensoftware.com/products/surfer/).

Direct velocity measurements carried out on moorings provide long-term velocity time series, which is important for studies of temporal variability of abyssal flows. However, the spatial resolution of such data is usually low due to high cost and logistical problems in deployment and recovery of moored deep-water stations. Comparing to the velocity measurements performed at moorings, the LADCP data provide fine cross-channel structure of the abyssal flow. Two LADCP sections across the Vema Channel on April 6, 2017 and October 26, 2018 are presented in Fig. 4. These sections captured two modes of the abyssal flow: very fast bottom current (Fig. 4a) with velocity up to 52 cm/s and moderate flow (Fig. 4c) with velocity up to 28 cm/s near the western wall of the channel. The SLA maps on the same dates show sea level differences which are in good agreement with LADCP sections: the SLA varies between 24 cm (Fig. 4b) and 16 cm (Fig. 4d). Spatial velocity structures show that variations observed on moorings are caused by acceleration and deceleration of the entire current rather than meandering the abyssal jets.

## Discussion

The observed correlation between abyssal velocity and surface sea level anomalies does not provide a dynamical explanation of the bottom current variability itself. However, it is well-known that the along-channel pressure gradient is the main force which moves abyssal waters from one deep ocean basin to another^35,36^. In the case of the Vema Channel, the relatively thick layer of dense AABW in the Argentine Basin and relatively thin layer of AABW in the Brazil Basin lead to the pressure difference which forces abyssal waters through the channel. It is natural to assume that variations in this pressure difference lead to variations in abyssal current velocity. Here we suggest that these abyssal pressure variations are related to sea level anomalies at the entrance to the Vema Channel. We assume that variations in the sea level over the entrance are connected with abyssal pressure variations (positive SLA indicate an increase in pressure over the entire water column including the abyssal layer while negative SLA indicate a decrease in pressure) which are sufficient for the observed changes in abyssal velocity. Unfortunately, there are no in situ temperature and salinity data along the studied abyssal flow which provide accurate data on abyssal pressure gradient because measurements at such depths are extremely rare. However, the observed correlation between the SLA and abyssal velocity indicate that the suggested mechanism is at least not negligible and should be considered in studies of abyssal ocean variability. Moreover, our study shows the exact locations where direct temperature and salinity measurements should be made for accurate evaluation of the abyssal pressure gradient and its influence on bottom currents; we hope that this is helpful for future expeditions which are repeatedly carried out in this region (see for example a review in ref^14^). One of the proofs that the intense mesoscale eddies can be related to the variations in abyssal pressure field is presented in direct LADCP measurements carried out in the Brazil-Malvinas Confluence zone^37,38^. In particular, velocities in the presence of mesoscale eddies reach 50 cm/s at a depth of 3000 m and deeper indicating significant variations in abyssal pressure gradients and corresponding velocity field.

The observed correlation provides another insight to the dynamics of the abyssal flow in the channel. One can assume that there are two types of pressure gradient which affect velocity: the local cross-slope pressure gradient and bulk meridional pressure gradient along the entire channel. Our study shows that the meridional pressure gradient is more important. The local cross-channel pressure gradient could be significant in case of the geostrophic flow, where changes in the lateral pressure gradient directly affect the geostrophic velocity. However, the abyssal flow in the Vema Channel is ageostrophic which makes this effect not as important as the along-channel pressure gradient. We also calculated correlation maps between abyssal velocity and local meridional and zonal SLA gradients (see supplementary Fig. S1 online) which do not show significant correlation and confirm the considerations given above.

It should be noted that there is no time lag between the surface and abyssal signals which are 400 km apart. The correlation calculated for shifted time series shows that changes in the sea level and abyssal velocity are most likely synchronous (see supplementary Fig. S2 online). This effect can be explained by the continuity equation (conservation of mass along the channel) which requires that the velocities in the entire channel should change synchronously.

The location of the high-correlated area is quite surprising at first glance. However, this effect may be simply explained because the area is located at the entrance to the Vema Channel where the abyssal current forms. Around this location, the velocity of abyssal flow changes from several millimeters per second (these are estimated values for the meridional AABW flow through wide ocean basins^39^) to approximately 30 cm/s on average. After this fast flow forms, it possesses significant amount of kinetic energy which cannot rapidly disappear in the channel. That is why local sea level anomalies over the mooring and downstream the channel cannot change the abyssal velocity. At the same time, the sea level anomalies at the entrance to the channel are important and negative SLA can be related even to the blocking events discussed above. These blocking events should rearrange the flow in the northern Argentine Basin; however, due to low velocities and short duration of the blocking events it is unlikely that these changes are measurable. Further measurements are needed for clarification of these processes.

Similar changes in the abyssal circulation including periods of reversal flows are found in other abyssal channels on the pathways of AABW spreading. For example, reversal currents are observed in the Kane Gap connecting deep basins in the Equatorial Atlantic^9^. Similar variability at synoptic time scales is found in the abyssal layer of the Northeast Pacific^11^ and Southwest Indian Ocean^40^. Repeated moorings deployed in the Vema Channel since 1979^28^ do not show significant long-term trends in the abyssal velocity. The blocking events are steadily observed in different mooring deployments^28^; usually the current stops and no obvious compensating rebounds are observed after these events.

## Concluding remarks

**Figure 5:**
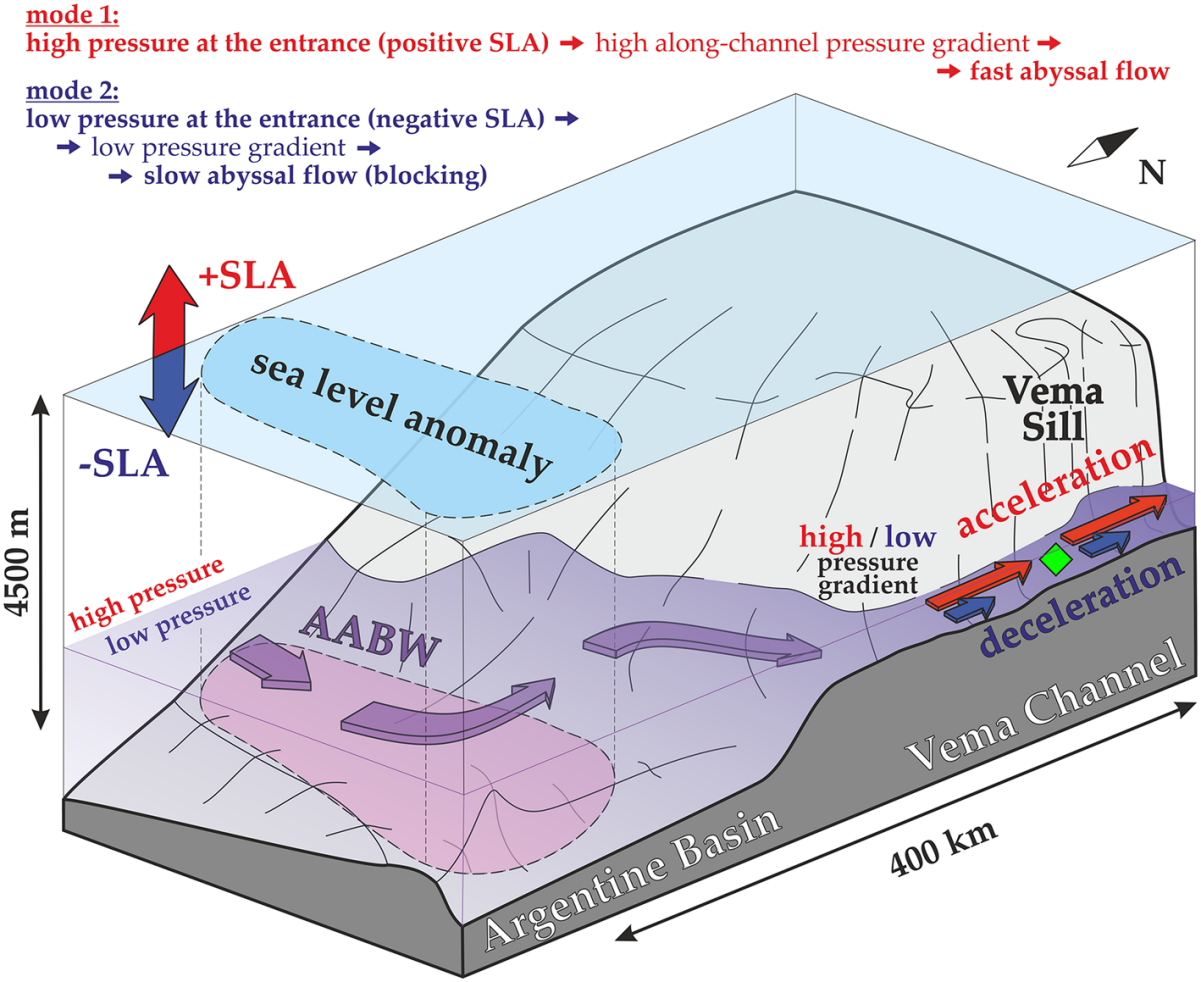
Schematic of influence of sea level anomalies in the northern Argentine Basin on the current in the Vema Channel. Red (blue) arrows indicate simultaneous increase (decrease) in the sea level and acceleration (deceleration) of the abyssal flow through the channel. The AABW flow pathway is shown by violet arrows. The green diamond indicates the location of mooring in the Vema Channel. Note significant difference in horizontal and vertical scales: the abyssal flow at approximately 4500 m depth in the Vema Channel is affected by sea level anomalies located 400 km upstream.

Our combined in situ and satellite data study has revealed a new dynamical mechanism of bottom current variability in the Vema Channel, which is likely at play in other key abyssal passages of the World Ocean. It has been also demonstrated that surface sea level anomalies can be related to the blocking events along pathways of AABW flow observed in many previous studies. We have considered here only the correlation between sea level anomalies and currents in the abyssal ocean layer; schematic of this connection is summarized in Fig. 5. Clearly, there are many other aspects affecting abyssal circulation at different temporal scales, including, for example, spatial differences in the location of the upper boundary of AABW layer^14^, tidal currents^40,41^, inertial motions inside the abyssal channels^6^, and long-term warming trends in the AABW layer^20^. In this work, we focused on the variability of abyssal currents on a synoptic scale, which can be affected by different processes and tried to find examples of clearly pronounced correlation between sea level anomalies and variations in the velocity of AABW flow in the Vema Channel. This study opens an interesting discussion about the importance of sea level anomalies on the entire three-dimensional ocean circulation including its deepest layers. The possible blocking of the current in the Vema Channel makes these processes important on a global scale and becomes especially important in a changing climate.

## Methods

### Satellite altimetry

Data on the Sea Level Anomalies (SLA) have been taken from the satellite altimetry gridded product^32^ available from Copernicus Marine Environment Monitoring Service (http://marine.copernicus.eu/). These data have a spatial resolution of 0.25° and a daily temporal resolution and includes data from all available altimeters at any given time. This product contains different types of altimetric data: Absolute Dynamic Topography (ADT), Sea Level Anomalies (SLA), and zonal and meridional components of computed surface geostrophic velocities. Though gridded altimetry data are provided with a daily resolution, the satellite revisit time is approximately 9.9 days or longer, depending on the platform. Thus, altimetry derived products do not capture SLA fluctuations typically shorter than the satellite revisit time.

### Moored velocity measurements

Velocity measurements in the abyssal ocean layer were performed at two moorings positioned 12.4 km apart. The moorings were deployed close to the eastern (31.25°S, 39.32°W) and western (31.25° S, 39.45° W) walls of the Vema Channel providing data on the cross-channel velocity structure. Two stages of measurements interrupted by a three-day service interval in the middle of the time series were carried out between 2003 and 2007. The total records last 3.4 years; sampling interval was set to two hours. Both acoustic current meters and Aanderaa meters with rotors were used for these measurements. A more detailed description of the data from moorings is presented in ref^29^. The map of correlation between the SLA and abyssal velocity time series was calculated separately for each point of the satellite altimetry grid. At each point, the Pearson correlation coefficient was calculated between 3.4-year long time series of SLA data at this point and moored velocity time series at 31.25° S, 39.32° W of the same length; the velocity data were taken from the recorder deployed at a depth of 4500 m.

### CTD and LADCP measurements

Distribution of meridional velocity component shown in Fig. 1c was measured at 8 oceanographic stations. The section was located across the Vema Channel at a latitude of 31° 12’ S. The velocity profiles were measured by the LADCP RDI WorkHorse Sentinel 300 kHz. Velocity measurements were accompanied by conductivity, temperature, and depth (CTD) measurements using the SBE SeaCat 19plus profiler. The measurements were performed from the sea surface almost to the ocean floor (5–7 m above the bottom) from the R/V “*Akademik Sergey Vavilov*” of the Russian Academy of Sciences. The raw LADCP data were processed using the LDEO Software version IX.10^42^; the TPXO9 model^43^ was used for estimating the barotropic tidal velocities and subtracting of these velocities from the measured data at the moment of measurements.

### Modeling of bottom circulation

Modeled bottom circulation presented in Fig. 1b is a result of numerical simulations performed using the Institute of Numerical Mathematics Ocean Model, INMOM^44^. This model was adjusted for simulations in the region of the Vema Channel with high spatial resolution^45,46^. The model is based on the full system of primitive thermo-hydrodynamic equations in spherical coordinates with the hydrostatic and Boussinesq approximations. The INMOM uses a vertical coordinate σ = σ(x,y,t) scaled by the depth of the ocean at the given point. Zero velocity and sea surface fields, and temperature and salinity fields from the climatological World Ocean Atlas were specified as initial conditions. These data were also used in a buffer zone with a width of 10 points at the liquid boundaries.

### Supplementary Information

Supplementary Figures.

## Data Availability

The satellite altimetry data are available at Copernicus Marine Environment Monitoring Service (http://marine.copernicus.eu/). The results of numerical simulations and LADCP data used in the publication are available in open access through Pangaea (https://doi.pangaea.de/10.1594/PANGAEA.907919). Velocity data from moored recorders in the Vema Channel are available at https://doi.org/10.17882/80927 and described in detail in ref^6,18^. CTD data from the Vema Channel are available in ref^47^ and can be downloaded from the public repository: http://dx.doi.org/10.17632/hh4hhn6ny8.1.

## References

[1] Mackinnon JA, Johnston TMS, Pinkel R (2008). Strong transport and mixing of deep water through the Southwest Indian Ridge. Nat. Geosci..

[2] Hansen B, Østerhus S (2000). North Atlantic Nordic Seas exchanges. Prog. Oceanogr..

[3] Rudnick DL (1997). Direct velocity measurements in the Samoan Passage. J. Geophys. Res. Oceans.

[4] Hansen B, Turrell WR, Østerhus S (2001). Decreasing overflow from the Nordic seas into the Atlantic Ocean through the Faroe Bank channel since 1950. Nature.

[5] Frey DI (2023). Multiple abyssal jets flowing into the Vema deep, Romanche fracture zone. J. Geophys. Res. Oceans.

[6] Morozov EG, Demidov AN, Tarakanov RY, Zenk W (2010). Abyssal Channels in the Atlantic Ocean: Water Structure and Flows.

[7] Zhou C, Xiao X, Zhao W (2023). Increasing deep-water overflow from the Pacific into the South China Sea revealed by mooring observations. Nat. Commun..

[8] Aleinik DI, Byshev VI, Neiman VG (2004). Variability of thermodynamic characteristics in the deep ocean. Dokl. Earth Sci..

[9] van Haren H, Morozov E, Gostiaux L, Tarakanov R (2013). Convective and shear-induced turbulence in the deep Kane Gap. J. Geophys. Res. Oceans.

[10] Hogg N, Siedler G, Zenk W (1999). Circulation and variability at the southern boundary of the Brazil Basin. J. Phys. Oceanogr..

[11] Connolly TP, McGill PR, Henthorn RG, Burrier DA, Michaud C (2020). Near-bottom currents at station M in the abyssal Northeast Pacific. Deep-Sea Res. II Top. Stud. Oceanogr..

[12] Tarakanov RY, Morozov EG, van Haren H, Makarenko NI, Demidova TA (2018). Structure of the deep spillway in the western part of the Romanche fracture zone. J. Geophys. Res. Oceans.

[13] Wüst, G. Schichtung und Zirkulation des Atlantischen Ozeans in *Wissenschaftliche Ergebnisse, Deutsche Atlantische Expedition auf dem Forschungs—und Vermessungsschiff “Meteor” 1925–1927* (ed. Defant, A.) Ch. 6 (Walter de Gruyter & Co., Berlin, 1936).

[14] Morozov EG, Tarakanov RY, Frey DI (2021). Bottom gravity currents and overflows in deep channels of the Atlantic Ocean observations, analysis, and modeling.

[15] Johnson GC (2008). Quantifying Antarctic bottom water and North Atlantic deep water volumes. J. Geophys. Res. Oceans.

[16] Zenk W, Speer KG, Hogg NG (1993). Bathymetry at the Vema sill. Deep Sea Res..

[17] Zenk W, Hogg NG (1996). Warming trend in Antarctic bottom water flowing into the Brazil Basin. Deep Sea Res. I.

[18] Speer KG, Zenk W (1993). The flow of Antarctic bottom water into the Brazil Basin. J. Phys. Oceanogr..

[19] Gamboa LAP, Buffler RT, Barker PF (1983). Initial Reports of the Deep Sea Drilling Project.

[20] Campos EJ (2021). Warming trend in Antarctic bottom water in the Vema channel in the South Atlantic. Geophys. Res. Lett..

[21] Locarnini RA (2018). World Ocean Atlas 2018, volume 1: Temperature. NOAA Atlas NESDIS.

[22] Frey DI, Morozov EG, Fomin VV, Diansky NA, Tarakanov RY (2019). Regional modeling of Antarctic bottom water flows in the key passages of the Atlantic. J. Geophys. Res. Oceans.

[23] Frey DI, Borisov DG, Fomin VV, Morozov EG, Levchenko OV (2022). Modeling of bottom currents for estimating their erosional-depositional potential in the Southwest Atlantic. J. Mar. Syst..

[24] Johnson GC, Cadot C, Lyman JM, McTaggart KE, Steffen EL (2020). Antarctic bottom water warming in the Brazil Basin: 1990s through 2020, from WOCE to deep Argo. Geophys. Res. Lett..

[25] Johnson GC, Lyman JM (2020). Warming trends increasingly dominates global ocean. Nat. Clim. Change.

[26] Purkey SG (2019). Unabated bottom water warming and freshening in the South Pacific Ocean. J. Geophys. Res. Oceans.

[27] Wadley M, Bigg G (1995). Abyssal channel flow in ocean circulation models with application to the Vema channel. J. Phys. Oceanogr..

[28] Zenk W, Visbeck M (2013). Structure and evolution of the abyssal jet in the Vema channel of the South Atlantic. Deep-Sea Res. II Top. Stud. Oceanogr..

[29] Zenk W (2008). Temperature fluctuations and current shear in Antarctic bottom water at the Vema sill. Prog. Oceanogr..

[30] Zenk W, Morozov EG (2007). Decadal warming of the coldest Antarctic bottom water flow through the Vema channel. Geophys. Res. Lett..

[31] Orúe-Echevarría D (2021). A view of the Brazil–Malvinas confluence, March 2015. Deep Sea Res. Part I Oceanogr. Res. Pap..

[32] Pujol MI (2016). DUACS DT2014: The new multi-mission altimeter data set reprocessed over 20 years. Ocean Sci..

[33] Mason E, Pascual A, Gaube P, Ruiz S, Pelegrí JL, Delepoulle A (2017). Subregional characterization of mesoscale eddies across the Brazil-Malvinas confluence. J. Geophys. Res. Oceans.

[34] Artana C (2018). Fronts of the Malvinas current system: Surface and subsurface expressions revealed by satellite altimetry, Argo floats, and Mercator operational model outputs. J. Geophys. Res. Oceans.

[35] Whitehead JA (1998). Topographic control of oceanic flows in deep passages and straits. Rev. Geophys..

[36] Pratt LJ, Whitehead JA (2007). Rotating Hydraulics: Nonlinear Topographic Effects in the Ocean and Atmosphere.

[37] Orúe-Echevarría D, Pelegrí JL, Machín F, Hernández-Guerra A, Emelianov M (2019). Inverse modeling the Brazil-Malvinas confluence. J. Geophys. Res. Oceans.

[38] Orúe-Echevarría D (2021). A view of the Brazil-Malvinas confluence, March 2015. Deep-Sea Res. I.

[39] Smythe-Wright D, Boswell S (1998). Abyssal circulation in the Argentine basin. J. Geophys. Res..

[40] Liao G (2016). Moored observation of abyssal flow and temperature near a hydrothermal vent on the Southwest Indian Ridge. J. Geophys. Res. Oceans.

[41] van Haren H (2015). A typical anticlockwise internal tidal motions in the deep ocean. Tellus A Dyn. Meteorol. Oceanogr..

[42] Visbeck M (2002). Deep velocity profiling using lowered acoustic Doppler current profiler: Bottom track and inverse solution. J. Atmos. Ocean Technol..

[43] Egbert GD, Erofeeva SY (2002). Efficient inverse modeling of barotropic ocean tides. J. Atmos. Ocean Technol..

[44] Zalesny VB (2010). Numerical simulation of large-scale ocean circulation based on the multicomponent splitting method. Russ. J. Numer. Anal. Math. Model..

[45] Frey DI, Fomin VV, Diansky NA, Morozov EG, Neiman VG (2017). New model and field data on estimates of Antarctic bottom water flow through the deep Vema channel. Dokl. Earth Sci..

[46] Frey DI, Fomin VV, Tarakanov RY, Diansky NA, Makarenko NI, Velarde MG, Tarakanov RY, Marchenko AV (2018). Bottom water flows in the Vema channel and over the Santos plateau based on the field and numerical experiments. The Ocean in Motion: Circulation, Waves, Polar Oceanography.

[47] Morozov EG, Frey DI (2021). CTD data over a repeated section in the Vema channel. Data Br..

