# Supplementary material for: Sea level anomalies affect the ocean circulation at abyssal depths
Source: Sci Rep. 2023 Nov 27;13:20829. doi: 10.1038/s41598-023-48074-9 (PMC10682481; doi:10.1038/s41598-023-48074-9)
Supplement: Supplementary file 1 — Supplementary Figures. [file 41598_2023_48074_MOESM1_ESM.docx]

*Scientific Reports*

Supplementary Information for

**Sea level anomalies affect the ocean circulation at abyssal depths**

D.I. Frey^1,2,3^, E.G. Morozov^1^, D.A. Smirnova^1^,

^1^Shirshov Institute of Oceanology, Russian Academy of Sciences, Moscow, Russia

^2^Marine Hydrophysical Institute, Russian Academy of Sciences, Sevastopol, Russia

^3^Moscow Institute of Physics and Technology, Dolgoprudny, Russia

Corresponding author: Dmitry Frey

Email: [dima.frey@gmail.com](mailto:dima.frey@gmail.com)

The file contains figures S1 and S2.


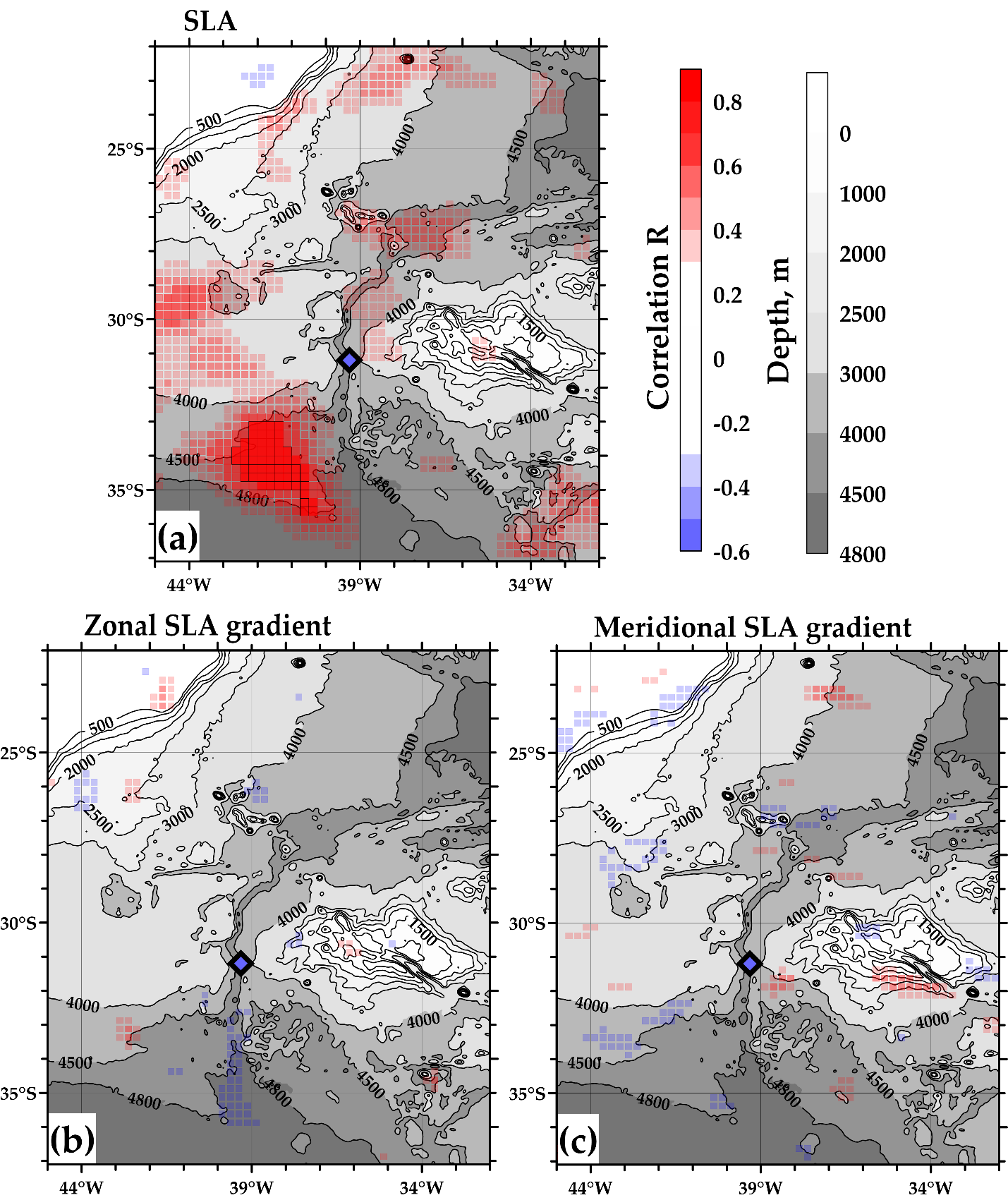


**Figure S1. Correlation maps between altimetry-derived variables and abyssal velocity.** Spatial correlation maps between moored velocity time series and (a) satellite altimetry sea level anomalies (SLA), (b) zonal SLA gradient, and (c) meridional SLA gradient. Zonal and meridional SLA gradients were calculated based on the sea level data at neighboring points of the standard altimetry 0.25° grid. The location of the mooring is shown by blue diamond. Only points with absolute value of correlation exceeding 0.3 are shown. The size of each grid point is 0.25° x 0.25°. The bottom topography is shown by shades of grey according to the GEBCO2022 database. The maps were generated using Golden Software Surfer version 18.1.186 (https://www.goldensoftware.com/products/surfer/).


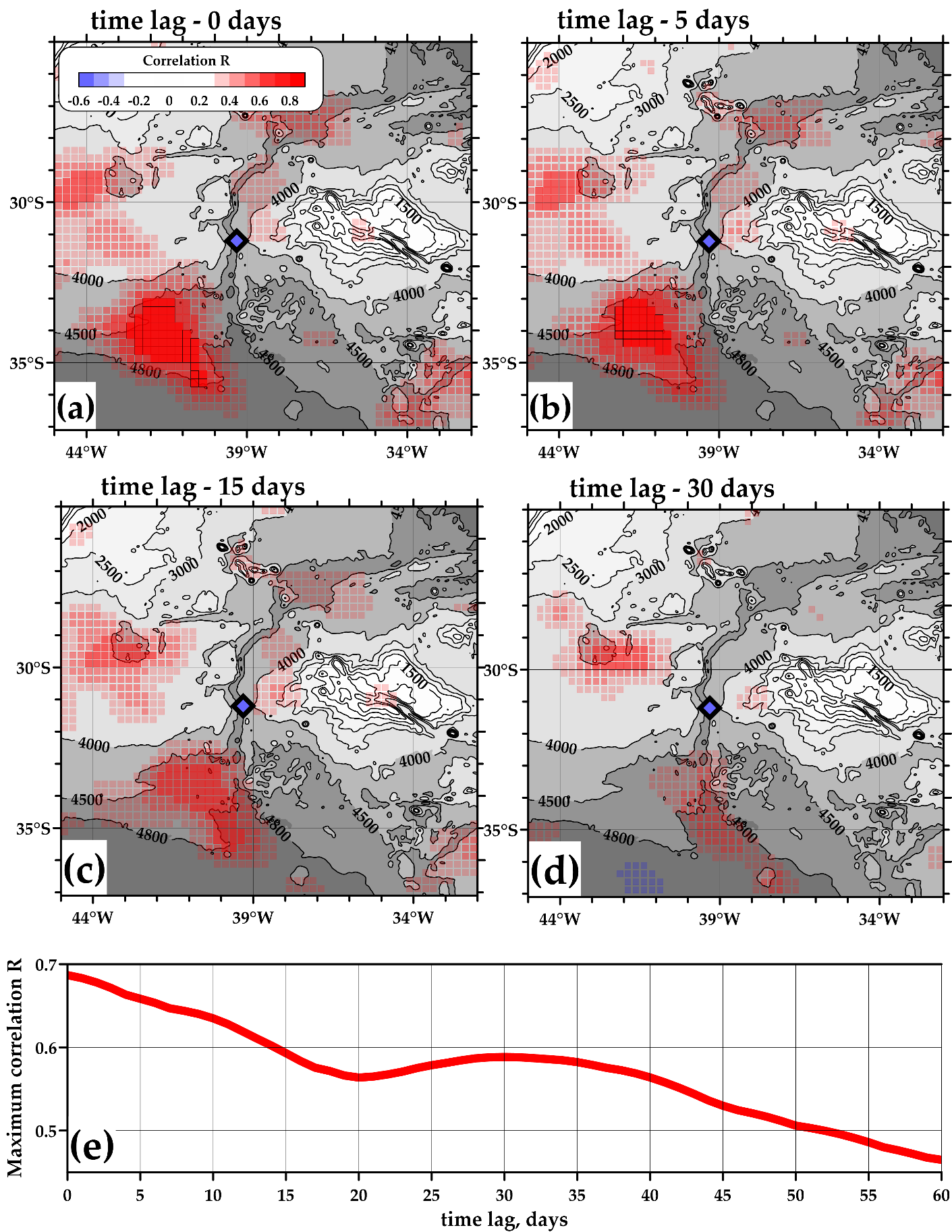


**Figure S2. Lagged spatial correlation between abyssal velocity and SLA data.** Maps (a,b,c,d) show the spatial correlation between SLA data at each point of the altimetry grid and shifted velocity time series. The velocity time series were shifted forward by (a) 0 days, (b) 5 days, (c) 15 days, and (d) 30 days. Panel (e) shows the maximum correlation (calculated as a maximum value over the entire maps) depending on the time lag in days. The location of the mooring is shown by blue diamond. Only points with absolute value of correlation exceeding 0.3 are shown. The size of each grid point is 0.25° x 0.25°. The bottom topography is shown by shades of grey according to the GEBCO2022 database. The maps were generated using Golden Software Surfer version 18.1.186 (https://www.goldensoftware.com/products/surfer/).
